# Development and validation of a nomogram to predict prognosis of patients with combined hepatocellular-cholangiocarcinoma after hepatic resection

**DOI:** 10.7150/jca.116790

**Published:** 2025-09-29

**Authors:** Rongqiang Liu, Sheng Wang, Yesheng Du, Tao Wen, Lei Xiang, Wenyuan Xie, Dewei Li, Hui Li

**Affiliations:** 1 Department of Hepatobiliary Pancreatic Tumor Center, Chongqing University Cancer Hospital, Chongqing, China.; 2 Department of Organ Transplantation, The Second Affiliated Hospital of Nanchang University, Nanchang, 330038, Jiangxi, China.; 3 Radiation Oncology Center, Chongqing University Cancer Hospital, School of Medicine, Chongqing University, Chongqing, China.

**Keywords:** combined hepatocellular carcinoma and cholangiocarcinoma, gamma-glutamyl transpeptidase to albumin ratio, nomogram, prognosis

## Abstract

**Background:** Combined hepatocellular carcinoma and cholangiocarcinoma (cHCC-CCA) is a rare primary liver cancer characterized by a low incidence but a poor prognosis. The purpose of the study was to develop a clinical prediction model utilizing non-invasive blood markers to effectively evaluate the prognosis of cHCC-CCA patients following hepatic resection.

**Methods:** The retrospective analysis was conducted on 125 patients with cHCC-CCA who underwent hepatic resection between April 2013 and October 2022. All cHCC-CCA patients were randomly assigned to the training group (n = 63) and the validation group (n =62). A nomogram based on patient clinical factors was established using cox regression analysis. Receiver operating characteristic curves (ROCs) were used to assess the predictive performance of the model. Calibration and decision curves were employed to evaluate the model's prediction accuracy and goodness of fit.

**Results:** Multivariate analysis revealed significant associations between lymphatic metastasis, microvascular invasion (MVI), gamma-glutamyl transpeptidase to albumin ratio (GAR), carcinoembryonic antigen (CEA), prothrombin time (PT), alpha-fetoprotein (AFP), hepatitis B virus (HBV), and overall survival. Based on these prognostic factors, a nomogram model was established and validated using the validation set. Calibration curves demonstrated good consistency in the 1-year, 3-year, and 5-year survival rates of patients. Additionally, the ROC analysis indicated the model's strong predictive ability, and the decision curves confirmed its clinical applicability.

**Conclusion:** This study successfully developed a nomogram model for predicting survival outcomes in patients with cHCC-CCA following hepatectomy.

## Introduction

Liver cancer as a significant global health burden, not only ranks as one of the most common malignancies, but also is the third leading cause of cancer-related mortality worldwide^1^. In 2018 alone, there were approximately 841,080 new cases and 781,631 deaths were attributed to liver cancer^2^. Among the primary liver cancer subtypes, hepatocellular carcinoma, intrahepatic cholangiocarcinoma, and combined hepatocellular carcinoma and cholangiocarcinoma (cHCC-CCA) are the main entities^3^. cHCC-CCA, a composite neoplasm comprising elements of both HCC and CCA, accounts for 0.4% to 14.2% of primary liver cancer cases^4^. This unique subtype predominantly affects middle-aged and older males, with viral hepatitis being a major risk factor in Asian populations, while the etiology in Western populations is multifactorial^5^. cHCC-CCA is characterized by insidious onset, rapid progression, and early metastatic potential. Radical hepatectomy is currently the primary treatment approach for cHCC-CCA^6^. However, despite effective hepatectomy, many patients experience tumor recurrence, leading to a 5-year survival rate of less than 30%^7^. Enhancing the prognosis of cHCC-CCA patients remains a challenging clinical dilemma. Thus, developing a more accurate predictive model for survival outcomes following hepatic resection in cHCC-CCA patients would aid clinicians in formulating optimal treatment strategies to improve overall survival rates.

Clinical prediction models incorporating various prognostic factors have been successfully employed to predict outcomes in numerous malignancies, including liver cancer and colorectal cancer^8^-^10^. Notably, Tang et al established a prediction model based on radiomics to assess overall survival outcome in cHCC-CCA patients^11^. While Wu et al developed a recurrence prediction model utilizing clinical information^12^. Nevertheless, despite these notable contributions, further investigations are warranted to deepen our understanding of cHCC-CCA and address existing knowledge gaps.

In this study, we collected comprehensive clinical information, including tumor markers, blood parameters, and pathological features, from patients diagnosed with cHCC-CCA. Based on identified prognostic risk factors, we aimed to establish a prognostic model based on non-invasive blood markers to accurately evaluate the survival outcomes of patients with cHCC-CCA following hepatic resection.

## Materials and Methods

### Patients

A total of 125 patients who were diagnosed with cHCC-CCA and underwent hepatic resection at Chongqing University Cancer Hospital between April 2013 and October 2022 were adopted in the study. Only patients with complete follow-up information were considered. The cHCC-CCA patients were randomly assigned to the training group and the validation group at a 1:1 ratio. The inclusion criteria: (1) confirmation of cHCC-CCA through pathology after hepatectomy, and (2) availability of complete postoperative follow-up data. The exclusion criteria included: (1) incomplete follow-up information; (2) presence of other tumors or extrahepatic metastasis; (3) receipt of preoperative anticancer treatments such as chemotherapy, radiotherapy, or immunotherapy; and (4) presence of other chronic diseases, such as uncontrolled diabetes, chronic kidney disease (stage ≥3), congestive heart failure. Patients with cirrhosis were included in this study. The Ethics Committee of Chongqing University Cancer Hospital approved the study and all patients signed informed consent forms. The study was implemented in accordance with the Declaration of Helsinki.

### Data collection and follow-up

All preoperative clinical information, including general patient characteristics, laboratory parameters, and tumor pathological features, was extracted from the hospital's electronic medical records. The collected data included gender, age, survival time, survival status, viral hepatitis infection, liver cirrhosis, ascites, tumor capsule status, blood loss, tumor number, microvascular invasion (MVI), tumor thrombus, satellite lesions, lymphatic metastasis, tumor size, alpha-fetoprotein (AFP) levels, Child-Pugh grade, American Joint Committee on Cancer (AJCC) stage, red blood cell (RBC) count, white blood cell (WBC) count, neutrophil count, lymphocyte count, hemoglobin (Hb) level, platelet count (PLT), macrophage count, total bilirubin (TBil) level, albumin (ALB) level, aspartate aminotransferase (AST) level, alanine aminotransferase (ALT) level, carbohydrate antigen 19-9 (CA19-9) level, carcinoembryonic antigen (CEA) level, alkaline phosphatase (ALP) level, gamma-glutamyl transpeptidase (GGT) level, prothrombin time (PT), plasma fibrinogen (FIB) level, and gamma-glutamyl transpeptidase to albumin ratio (GAR). GAR was calculated by dividing the GGT count by the ALB count. Follow-up assessments were initiated 3 months after surgery and conducted at least twice a year during the first 2 years. The follow-up period ended in October 2022. Follow-up information included blood biochemical tests, abdominal dynamic enhanced computed tomography, magnetic resonance imaging, or PET-CT scans. Overall survival (OS) was recognized as the time from hepatectomy to death or the last follow-up in patients with cHCC-CCA.

### Statistical analysis

All statistical analyses were implemented through R software (version 4.0.2). All cHCC-CCA patients were randomly allocated into the training group and the validation group at a ratio of approximately 1:1. Pearson's correlation coefficient was applied to analyze the correlation between variables, and a heatmap was generated. The Kaplan-Meier methods were employed to construct survival curves. The optimal cut-off values for continuous variables were determined using X-tile software, which identifies the best stratification threshold based on survival outcomes. The independent samples t-test was applied for normally distributed continuous variables, while non-normally distributed continuous data was analyzed by wilcoxon rank-sum test. The chi-square test was used for categorical variables. Cox regression models were utilized to analyze the prognostic risk factors. Variables with p value < 0.05 in the multivariate analysis were incorporated into the nomogram construction. Receiver operating characteristic curves (ROC) were adopted to assess the predictive performance of the model^13^. Finally, decision curves were employed to evaluate the clinical utility of the model^14^. P-value less than 0.05 was considered statistically significant.

## Results

### The best cut-off value for GAR

**Figure 1:**
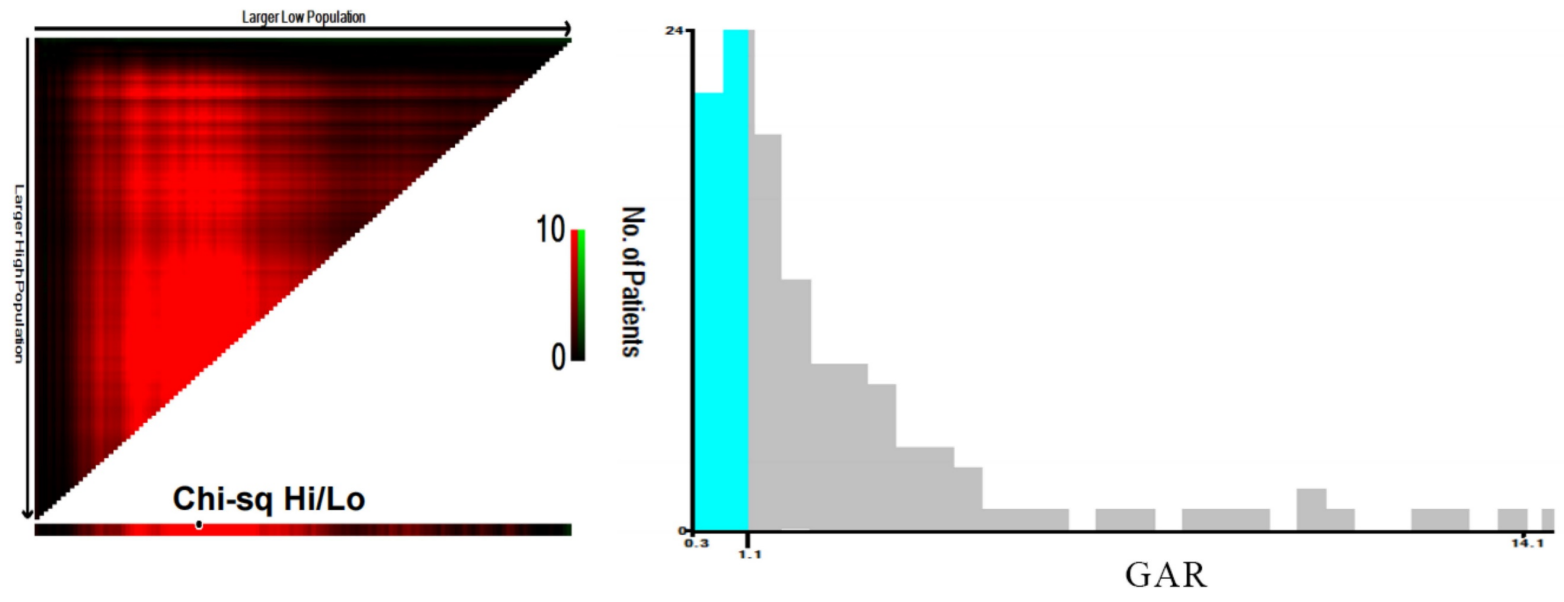
** Determination of the optimal cut-off value for the GAR using X-tile software.** The X-tile analysis identified the optimal cut-off value of GAR that best stratifies patients with combined hepatocellular-cholangiocarcinoma (cHCC-CCA) after hepatic resection based on overall survival. The figure illustrates the best division point along with corresponding chi-square values and survival curves.

X-Tile software was utilized to analyze the optimal cut-off value for the continuous variable, GAR, and convert it into a binary variable. The optimal cut-off value for GAR was determined to be 1.14 (Figure 1).

### Basic patient information

**Figure 2:**
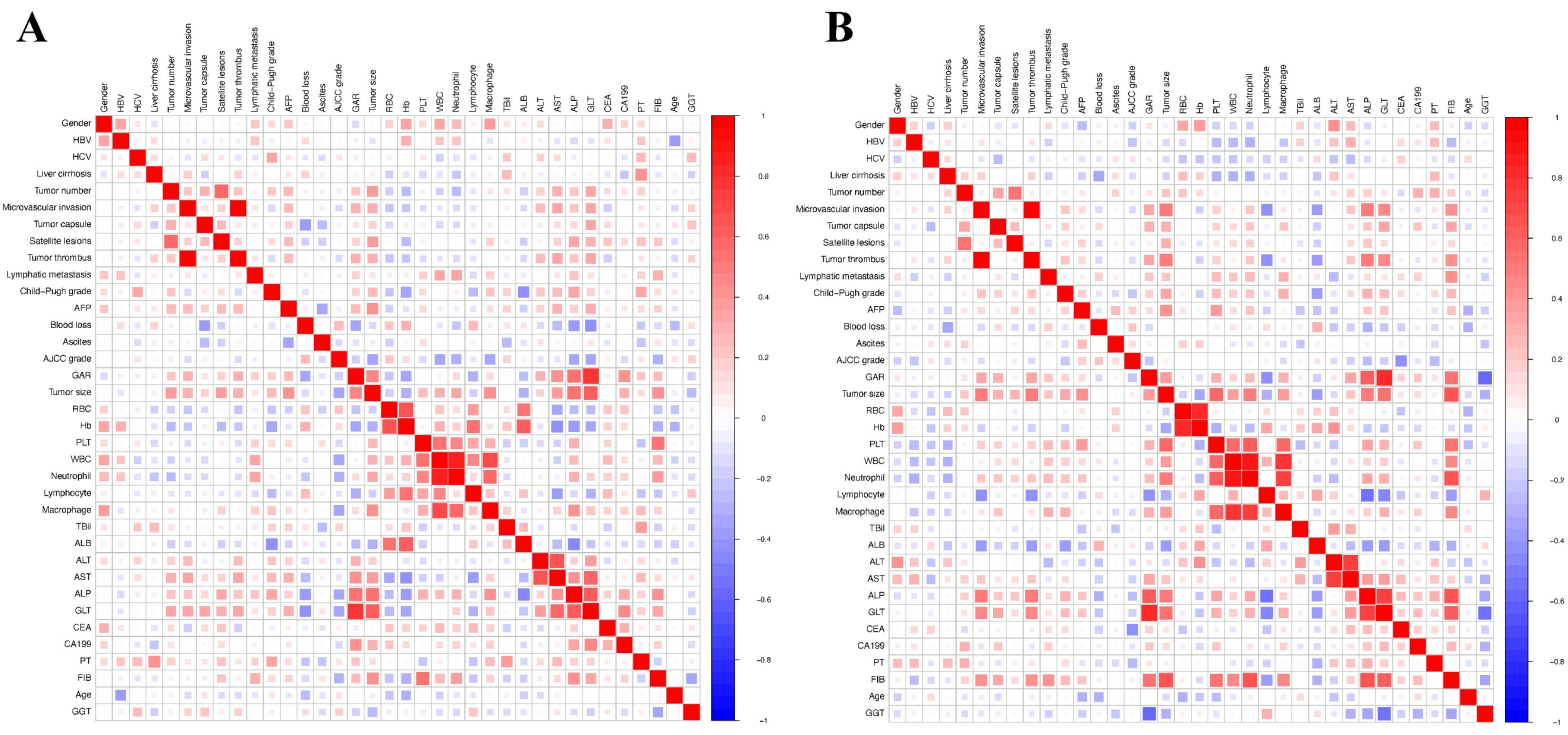
** Heatmaps showing the correlation between clinical and laboratory variables.** (**A**) Training cohort. (**B**) Validation cohort. Pearson correlation coefficients are represented using a color gradient, with red indicating a positive correlation and blue indicating a negative correlation. The heatmaps help visualize potential collinearity or associations among variables considered in the Cox regression model.

A total of 125 patients with cHCC-CCA who underwent radical hepatectomy and had complete follow-up data were included in the study. Of these patients, 105 patients were male. The majority of patients were over 50 years old, with an average age of 51.34 ± 10.73. Microvascular invasion (MVI) was observed in 42 patients (33.6%), while lymphatic metastasis was present in 16 patients (12.8%). Elevated AFP levels (> 400 ng/mL) were observed in 36 patients (28.8%). Among the patients, 81 patients were infected with HBV, while only 4 patients were infected with HCV. Cirrhosis was found in a significant proportion of patients (68%). Furthermore, 58 patients (46.4%) had two or more tumors. The division was based on a 1:1 ratio, resulting in 63 patients in the training group and 62 patients in the validation group. Baseline clinical features showed no significantly discrepancy between the two groups. The median overall survival was 20.6 months in the training group and 21.4 months in the validation group. Table 1 presented the basic patient characteristics. Additionally, spearman correlation analysis was conducted on the clinical information of patients in the two groups. Positive correlations were indicated by red, while negative correlations were denoted by blue (Figure 2).

### Univariate analysis and multivariate cox regression

**Figure 3:**
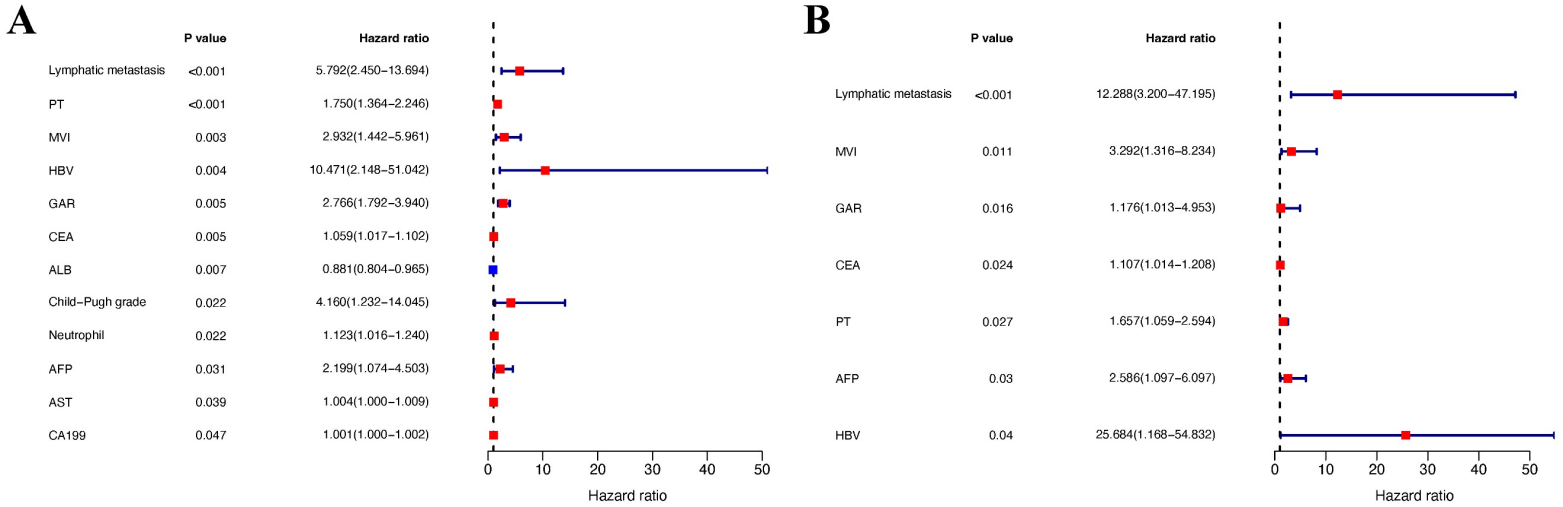
** Forest plots of Cox proportional hazards regression analyses for overall survival in the training cohort.** (**A**) Univariate analysis. (**B**) Multivariate analysis. Hazard ratios (HRs) and 95% confidence intervals (CIs) are shown for each variable. Significant prognostic factors identified in the univariate analysis were further included in the multivariate analysis to identify independent predictors of survival.

**Figure 4:**
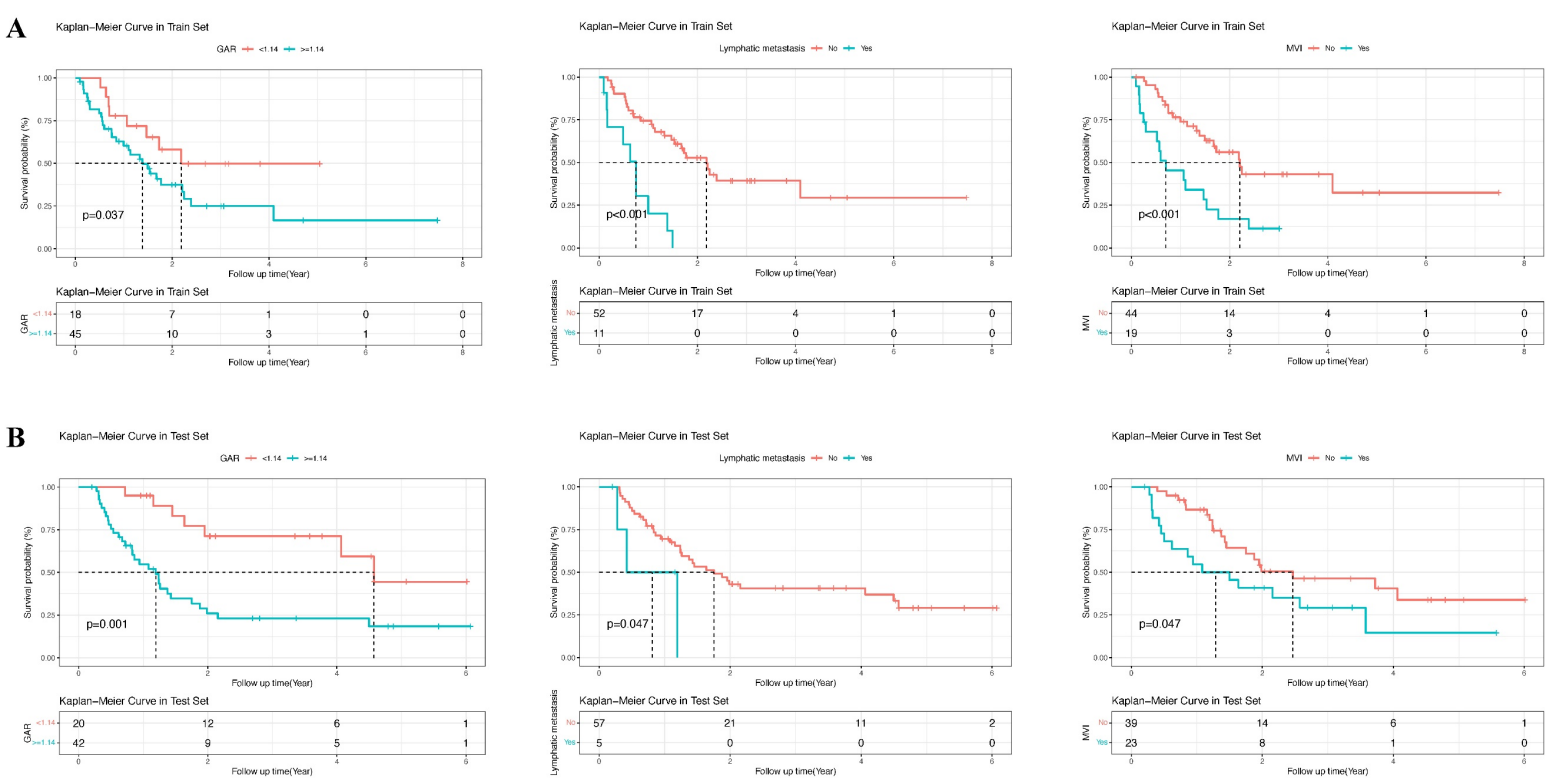
** Kaplan-Meier survival curves for overall survival stratified by GAR-based risk groups.** (**A**) Training cohort. (**B**) Validation cohort. Patients were divided into high-risk and low-risk groups according to the GAR cut-off value. The log-rank test was used to compare survival differences between the two groups, demonstrating the prognostic value of GAR.

In the training group, univariate analysis was initially performed, followed by multivariate analysis of variables with a P-value < 0.05 to identify the prognostic risk factors. Univariate analysis revealed that lymphatic metastasis, PT, MVI, HBV, GAR, ALB, AST, Child-Pugh grade, neutrophils, CEA, AFP, and CA19-9 were significant risk factors. These variables were then included in the multivariate analysis, which demonstrated that lymphatic metastasis, MVI, GAR, PT, AFP, CEA, and HBV were independent prognostic risk factors for patients with cHCC-CCA (P-value < 0.05) (Table 2). The forest plots were used to depict the results (Figure 3). Additionally, survival curves were described for the binary variables of the independent risk factors, and the results displayed that the P-values for the three binary variables (MVI, lymphatic metastasis, and GAR) were all less than 0.05 (P-value < 0.05) (Figure 4).

### Construction and validation of the nomogram

**Figure 5:**
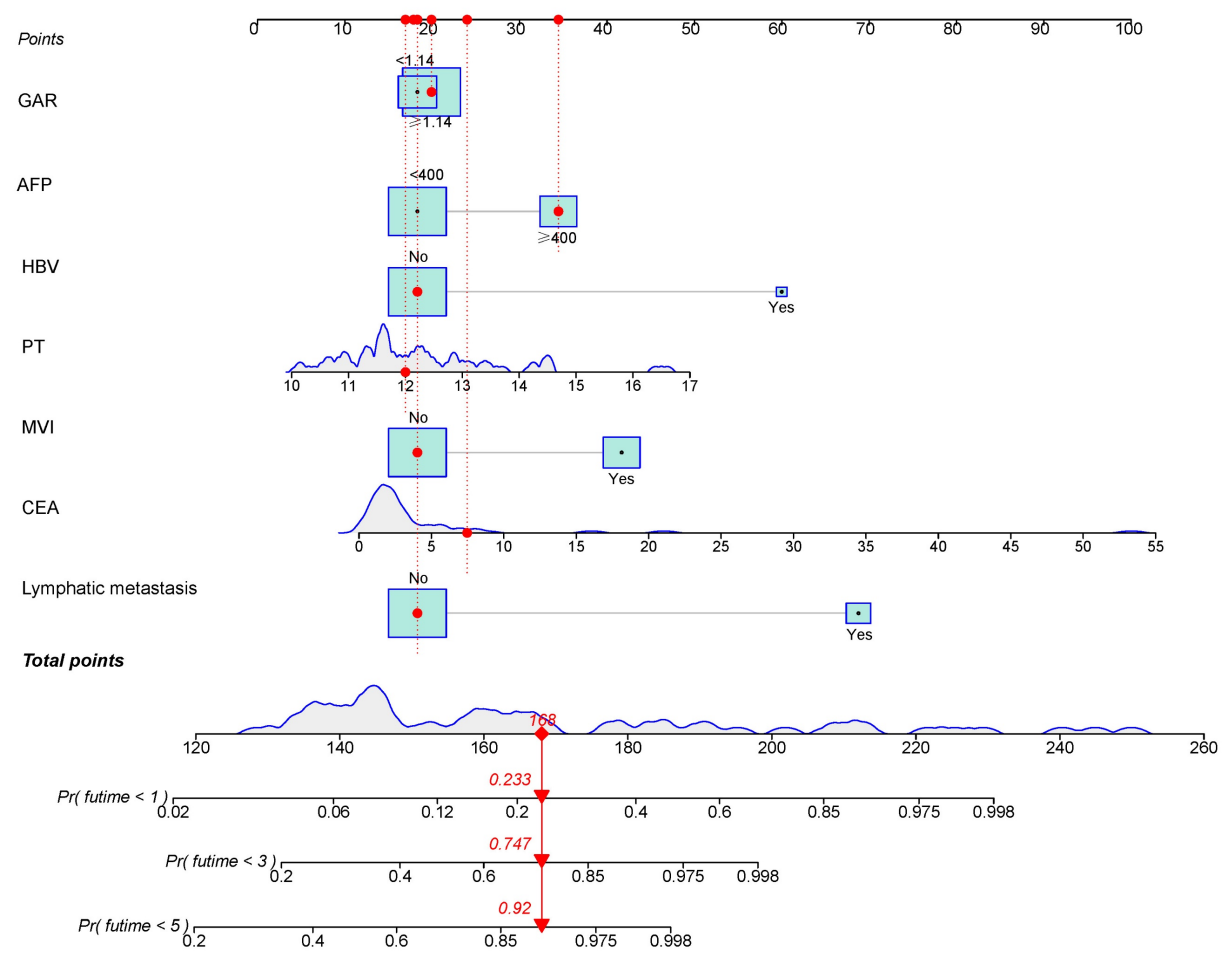
** Nomogram for predicting 1-, 3-, and 5-year overall survival in patients with cHCC-CCA after hepatic resection.** The nomogram incorporates independent prognostic factors identified in multivariate Cox analysis. Each variable corresponds to a specific score, and the total score predicts individual survival probabilities at 1, 3, and 5 years.

**Figure 6:**
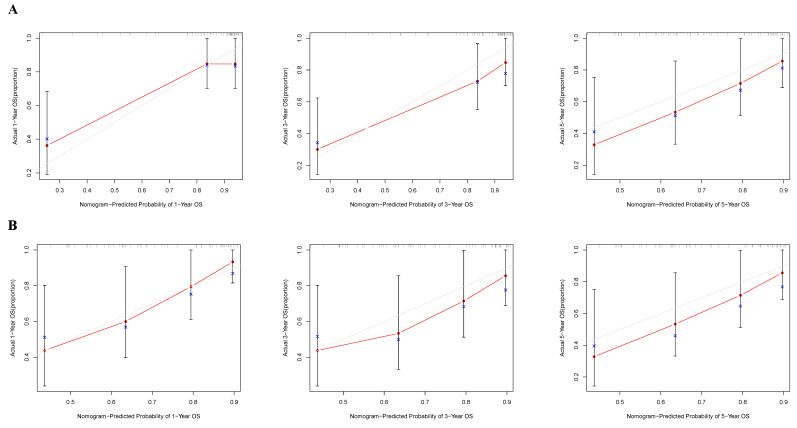
** Calibration curves assessing the predictive performance of the nomogram.** (**A**) Training cohort. (**B**) Validation cohort. The calibration plots compare the predicted survival probabilities from the nomogram with the observed outcomes at 1, 3, and 5 years. The 45-degree dashed line indicates perfect prediction, and close alignment with this line indicates good model calibration.

**Figure 7:**
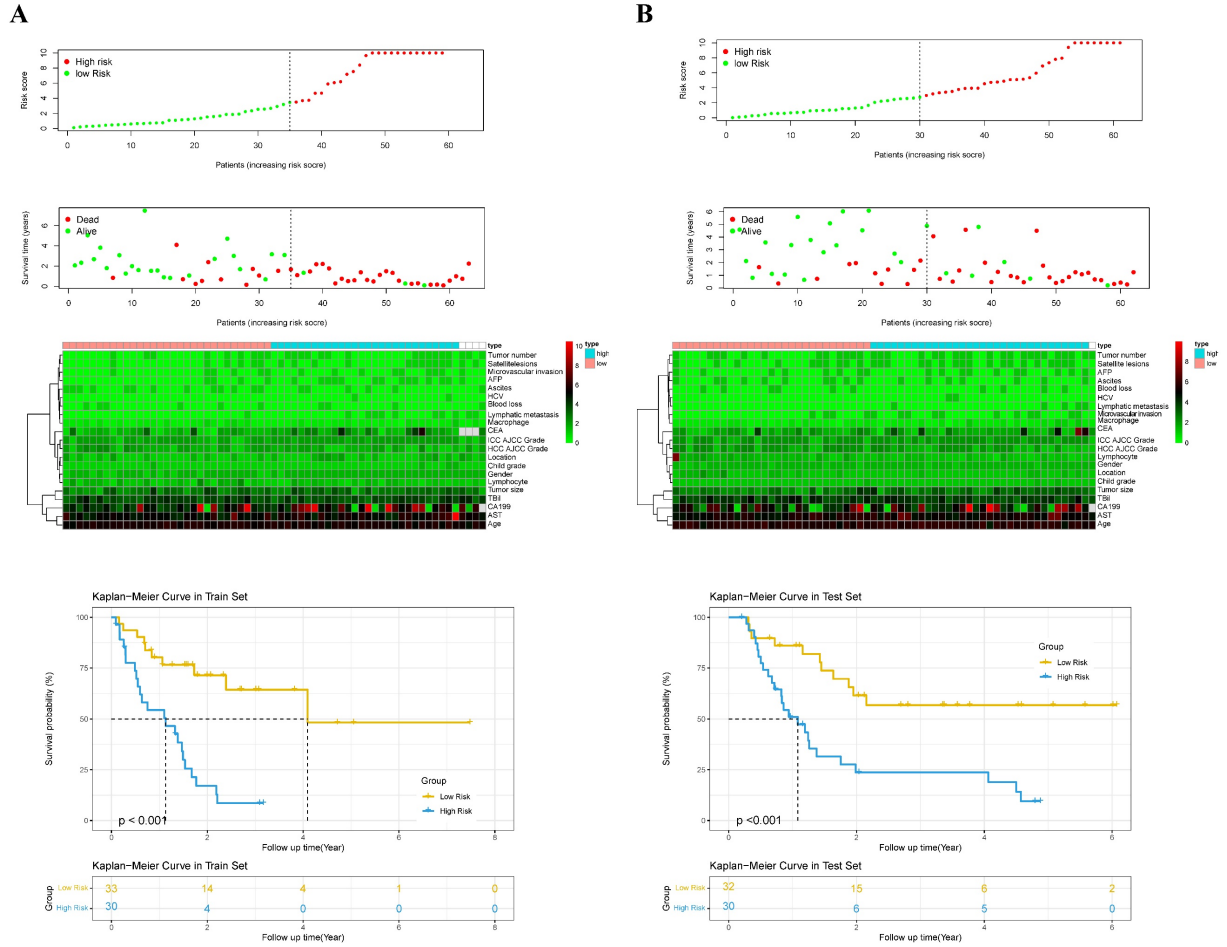
** Kaplan-Meier survival curves based on nomogram-derived risk scores.** (**A**) Training cohort. (**B**) Validation cohort. Patients were stratified into high- and low-risk groups using the median risk score from the nomogram. The survival curves demonstrate significant survival differences between groups, indicating the discriminatory power of the risk model.

According to the independent prognostic factors, a prognostic model for overall survival (OS) in cHCC-CCA patients was constructed (Figure 5). The corresponding score for each prognostic index can be determined from the column graph. The sum of the eight scores yielded the total score, which corresponded to the predicted OS rates for patients at 1, 3, and 5 years. To further evaluate and validate the nomogram, calibration curves were plotted for 1, 3, and 5-year survival rates in both the training and validation groups. The results demonstrated that predicted survival rates were in good agreement with actual survival rates (Figure 6). Furthermore, prognostic risk maps, risk heat maps, and survival time plots were generated to analyze the prognostic risk of cHCC-CCA patients, revealing that patients in the high-risk group had more unfavorable prognostic outcome (Figure 7).

### Predictive power and discrimination of the model

**Figure 8:**
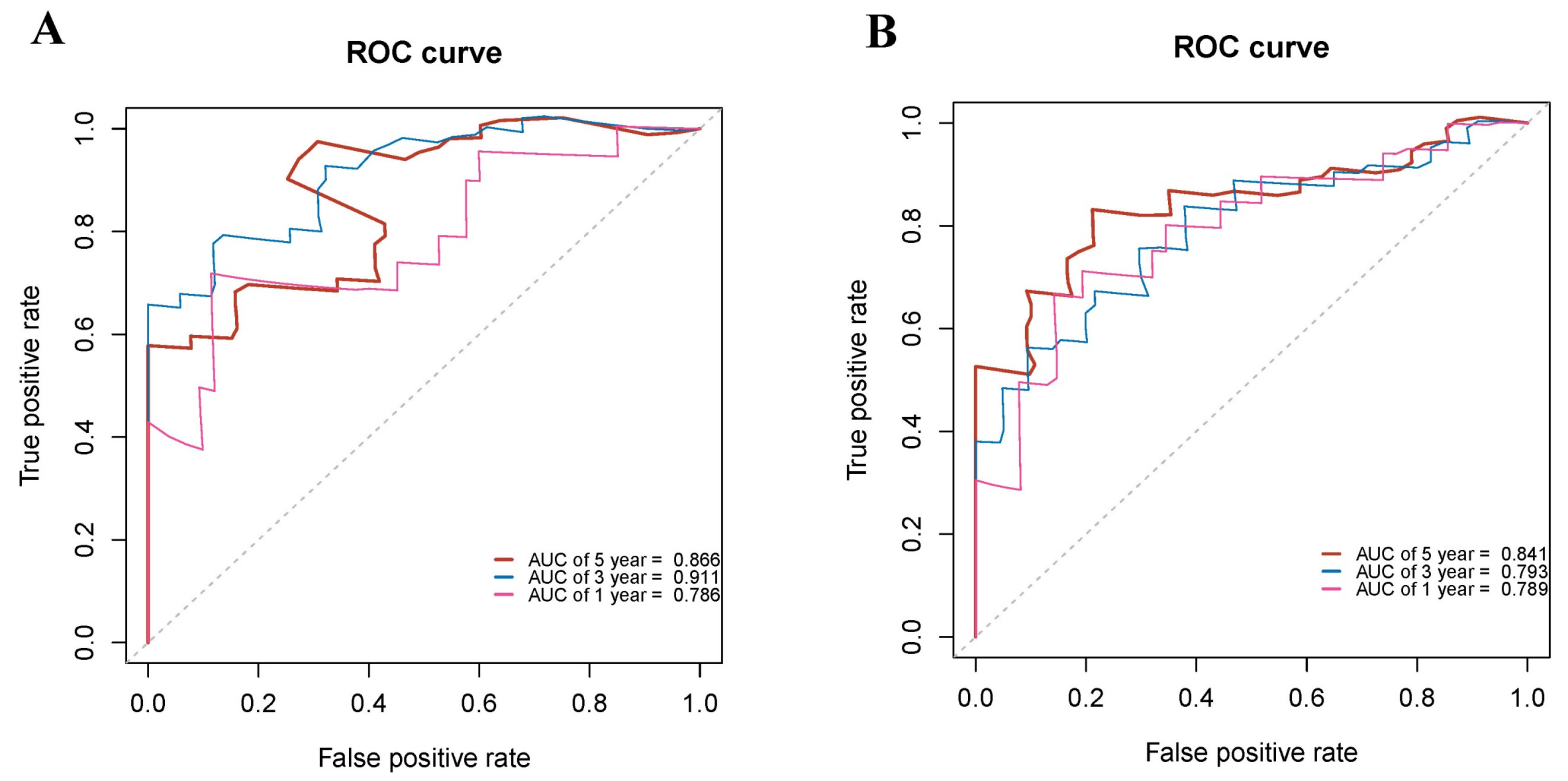
** Receiver operating characteristic (ROC) curves evaluating the performance of the nomogram in predicting overall survival.** (**A**) ROC curves for 1-, 3-, and 5-year survival in the training cohort. (**B**) ROC curves for 1-, 3-, and 5-year survival in the validation cohort. The area under the curve (AUC) values reflects the predictive accuracy of the nomogram at each time point.

**Figure 9:**
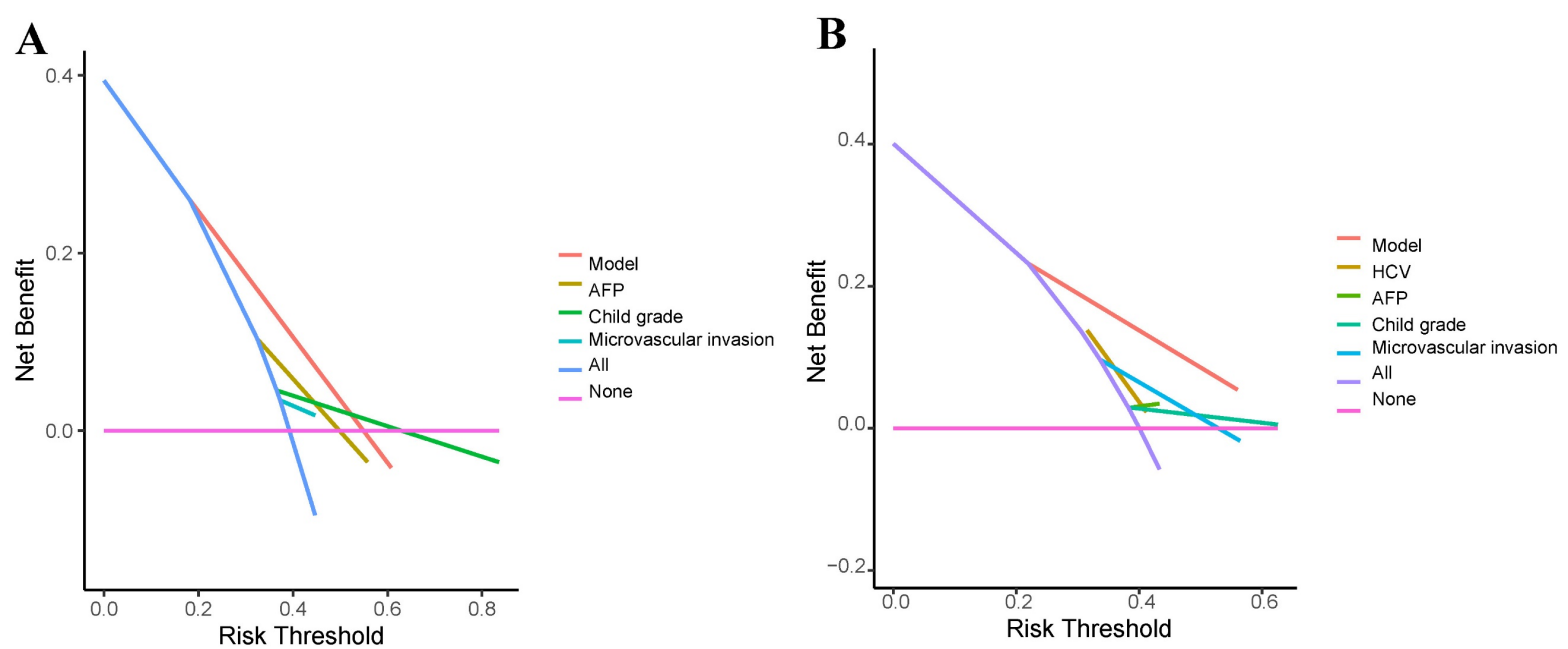
** Decision curve analysis (DCA) for the clinical utility of the nomogram.** (**A**) Training cohort. (**B**) Validation cohort. DCA evaluates the net clinical benefit of using the nomogram across a range of threshold probabilities, comparing it to default strategies (treat-all or treat-none). The higher net benefit across relevant thresholds indicates the model's potential utility in clinical decision-making.

To analyze the accuracy of the prediction model, ROC curves were plotted for different survival times. In the training group, the areas under the ROC curve of 1, 3, and 5-year OS were 0.786, 0.911, and 0.866, respectively (Figure 8A). In the validation group, the p areas under the ROC curve of 1, 3, and 5-year OS were 0.789, 0.793, and 0.841, respectively (Figure 8B). Lastly, decision curves were generated in two groups, demonstrating that the prediction model had higher accuracy compared to individual clinical variables (Figure 9).

## Discussion

Liver cancer is a significant global health concern, and cHCC-CCA is a subtype associated with limited research and clinical consensus regarding its clinical characteristics, treatment strategies, and prognosis. The low incidence of cHCC-CCA and varying classification criteria have contributed to the limited knowledge in this area. Previous studies have identified several factors, such as tumor differentiation, tumor size, CA19-9, AFP, and Child-Pugh score, as the prognostic risk factors in cHCC-CCA patients^15^, ^16^. However, the variables included in these studies were limited. In our study, we built a nomogram incorporating different variables to evaluate the prognostic outcome of patients with cHCC-CCA and assessed its performance in the training group. The calibration curves, ROC curves, and decision curves demonstrated the favorable prognostic predictive capability of the model.

cHCC-CCA is more commonly observed in men, and our study revealed that male patients accounted for 80% of the cohort^2^. In terms of viral etiology, 78% of patients were infected with HBV, while only 0.03% were infected with HCV. Cumulative studies have confirmed the important role of HBV infection in the progression of cHCC-CCA^5^. Regarding tumor markers, elevated AFP levels were observed in 28.8% of patients, which is lower than the previously reported 50%^17^. Furthermore, our study observed a higher proportion of patients with cirrhosis (68%) compared to previous studies, possibly due to the inclusion of predominantly HBV-infected patients^18^.

Through univariate and multivariate analyses, we identified lymphatic metastasis, PT, MVI, HBV, GAR, ALB, AST, Child-Pugh grade, CEA, AFP, and CA19-9 as independent prognostic risk factors for patients with cHCC-CCA. The findings were consistent with previous researches that have implicated lymph node metastasis, MVI, CEA, and AFP in the prognosis of cHCC-CCA^19^, ^20^.

Radical resection remains the primary treatment modality for cHCC-CCA patients. Previous studies have shown that radical resection is correlated with significantly improved survival outcome compared to palliative resection or non-surgical treatment^21^. In our study, the median survival time after radical resection was 21 months, which surpassed the mean survival time reported in previous research. Due to the similarities of cHCC-CCA with HCC in respect of portal and hepatic vein invasion and similarities with ICC in lymph node metastasis, radical resection, negative tumor margin and lymph node dissection are essential for achieving better outcomes^22^.

The prognostic significance of GAR, a composite marker consisting of GGT and ALB, was demonstrated in our study. GAR has been previously used as a stratification tool in chronic hepatitis B patients and has emerged as a prognostic risk factor in HCC, ICC, and pancreatic cancer patients following radical resection^23^-^25^. High GGT levels are related to increased tumor cell proliferation, invasion, and metastasis due to elevated reactive oxygen species and oxidative stress^26^. Serum GGT levels have also been proposed as prognostic indicators in various cancers^27^. On the other hand, ALB, produced by the liver, reflects nutritional status and liver function. Hypoalbuminemia is associated with immune cell dysfunction and immune evasion, leading to poorer survival outcomes in cancer patients^28^. GAR provides a simple and effective means of evaluating the prognosis of tumor patients, reflecting both malnutrition and inflammation. Lymphocyte-to-CRP ratio is a promising indicator and has been proven to have potential prognostic value in HCC and ICC^29^. Lymphocyte-to-CRP ratio should be considered in future comparative or integrative prognostic models.

Additionally, we identified PT as an independent prognostic risk indicator for cHCC-CCA patients after hepatectomy. Prolonged PT has been associated with adverse overall survival in HCC, increased recurrence risk in colorectal cancer, and poor prognosis in cholangiocarcinoma patients^30^, ^31^.

The study had a few disadvantages. It was a single-center retrospective study, which may introduce bias. Furthermore, the insufficient sample size may limit the generalizability and reliability of the findings. Additionally, our study predominantly included cHCC-CCA patients with HBV-infected, and further validation was needed to assess the applicability of the model for non-HBV-infected cHCC-CCA patients.

## Conclusions

We successfully developed a nomogram incorporating clinical risk factors to evaluate the overall survival outcomes of cHCC-CCA patients after hepatic resection. This prognostic nomogram provides a practical tool for stratifying cHCC-CCA patients into different risk categories based on readily available clinical and pathological parameters. In clinical settings, it can assist surgeons and oncologists in preoperative counseling, postoperative surveillance planning, and individualized decision-making for adjuvant therapies. For example, patients identified as high risk by the model may benefit from more intensive follow-up or consideration for early adjuvant treatments.

## Figures and Tables

**Table 1:** Baseline characteristics of patients with cHCC-CCA in the training and validation groups.

Variables	Total (n = 125)	Training (n = 63)	Validation (n = 62)	P-value
Gender, n (%)				0.838
Female	20 (16)	11 (17)	9 (15)	
Male	105 (84)	52 (83)	53 (85)	
HBV, n (%)				0.903
No	44 (35)	23 (37)	21 (34)	
Yes	81 (65)	40 (63)	41 (66)	
HCV, n (%)				1
No	121 (97)	61 (97)	60 (97)	
Yes	4 (3)	2 (3)	2 (3)	
Liver cirrhosis, n (%)				1
No	40 (32)	20 (32)	20 (32)	
Yes	85 (68)	43 (68)	42 (68)	
Tumor number, n (%)				0.181
Single tumor	67 (54)	38 (60)	29 (47)	
Multiple tumors	58 (46)	25 (40)	33 (53)	
Microvascular invasion;, n (%)				0.528
No	83 (66)	44 (70)	39 (63)	
Yes	42 (34)	19 (30)	23 (37)	
Tumor capsule, n (%)				1
No	43 (34)	22 (35)	21 (34)	
Yes	82 (66)	41 (65)	41 (66)	
Satellite lesions, n (%)				0.122
No	78 (62)	44 (70)	34 (55)	
Yes	47 (38)	19 (30)	28 (45)	
Lymphatic metastasis, n (%)				0.192
No	109 (87)	52 (83)	57 (92)	
Yes	16 (13)	11 (17)	5 (8)	
Child-Pugh grade, n (%)				1
A	118 (94)	59 (94)	59 (95)	
B	7 (6)	4 (6)	3 (5)	
AFP, n (%)				1
<400	89 (71)	45 (71)	44 (71)	
≥400	36 (29)	18 (29)	18 (29)	
Ascites, n (%)				0.888
No	89 (71)	44 (70)	45 (73)	
Yes	36 (29)	19 (30)	17 (27)	
AJCC grade, n (%)				0.73
I	14 (11)	7 (11)	7 (11)	
II	21 (17)	11 (17)	10 (16)	
III	90 (72)	45 (71)	45 (73)	
GAR, n (%)				0.8
<1.14	38 (30)	18 (29)	20 (32)	
≥1.14	87 (70)	45 (71)	42 (68)	
Tumor size, Median (Q1,Q3)	6 (4, 8)	6.1 (4, 8.6)	5.5 (3.82, 7.65)	0.259
Age, Mean ± SD	51.34 ± 10.73	52.05 ± 10.25	50.63 ± 11.23	0.462
RBC, Mean ± SD	4.64 ± 0.47	4.67 ± 0.49	4.62 ± 0.45	0.576
Hb, Mean ± SD	140.98 ± 15.55	139.22 ± 16.61	142.76 ± 14.3	0.204
PLT, Median (Q1,Q3)	144 (94, 184)	144 (99, 194)	143 (92.5, 177.75)	1
WBC, Median (Q1,Q3)	5.75 (4.73, 7.06)	5.65 (4.74, 7.14)	5.86 (4.74, 6.98)	0.537
Neutrophil, Median (Q1,Q3)	3.5 (2.9, 4.62)	3.51 (2.91, 4.62)	3.49 (2.88, 4.61)	0.838
Lymphocyte, Median (Q1,Q3)	1.36 (1.1, 1.81)	1.23 (0.98, 1.81)	1.42 (1.18, 1.8)	0.098
Macrophage, Median (Q1,Q3)	0.41 (0.3, 0.51)	0.37 (0.3, 0.51)	0.43 (0.32, 0.5)	0.305
TBil, Median (Q1,Q3)	13 (9.7, 17)	13 (9.55, 16.75)	12.95 (9.85, 16.85)	0.634
ALB, Median (Q1,Q3)	42.3 (38.9, 44.3)	42.3 (40.25, 44.85)	42.2 (38.38, 43.9)	0.392
ALT, Median (Q1,Q3)	34 (26, 48)	34 (25.5, 49)	34 (26.25, 48)	0.795
AST, Median (Q1,Q3)	36 (28, 48)	36 (31, 54)	36 (27.25, 46.75)	0.662
GGT, Median (Q1,Q3)	53 (44, 132)	60.5 (73.5, 125)	46 (28, 66.5)	0.086
ALP, Median (Q1,Q3)	101 (75, 135)	102 (77, 129.5)	99 (72.25, 135.75)	0.68
CEA, Median (Q1,Q3)	2.19 (1.52, 3.3)	2.2 (1.51, 3.56)	2.19 (1.56, 3.29)	0.933
CA19-9, Median (Q1,Q3)	27.3 (13.98, 72.75)	29.88 (15.29, 87.42)	21.73 (11.33, 51.6)	0.174
PT, Median (Q1,Q3)	11.9 (11.35, 12.8)	11.95 (11.33, 12.88)	11.9 (11.4, 12.8)	0.887
FIB, Median (Q1,Q3)	3.12 (2.41, 3.9)	3.2 (2.5, 3.89)	2.98 (2.14, 4.3)	0.379
Median OS (months)	21	20.6	21.4	0.82

**Abbreviations:** RBC, red blood cell; Hb, hemoglobin; PLT, platelets; WBC, white blood cell; TBil, total bilirubin; ALB, albumin; ALP, alkaline phosphatase; GGT, gamma-glutamyl transpeptidase; PT, prothrombin time; FIB, fibrinogen; CA19-9, carbohydrate antigen 19-9; GAR, gamma-glutamyl transpeptidase to albumin ratio.

**Table 2:** Univariate and multivariate cox regression analysis

Characteristics	Category	Univariate analysis		Multivariate analysis	
		HR (95% CI)	P-value	HR (95% CI)	P-value
AFP	<400	Ref	Ref	Ref	Ref
	≥400	2.199 ( 1.074 - 4.503 )	0.031	2.586 ( 1.097 - 6.097 )	0.03
Ascites	No	Ref	Ref		
	Yes	0.462 ( 0.199 - 1.07 )	0.072		
Tumor capsule	No	Ref	Ref		
	Yes	0.992 ( 0.488 - 2.014 )	0.982		
Child-Pugh grade	A	Ref	Ref	Ref	Ref
	B	4.16 ( 1.232 - 14.045 )	0.022	0.12 ( 0.001 - 12.548 )	0.372
Gender	Female	Ref	Ref		
	Male	1.458 ( 0.561 - 3.788 )	0.439		
GAR	<1.14	Ref	Ref	Ref	Ref
	≥1.14	2.766 ( 1.792 - 3.94 )	0.005	1.176 ( 1.013 - 4.953 )	0.016
Blood loss	<500ml	Ref	Ref		
	≥500ml	1.372 ( 0.59 - 3.192 )	0.462		
HCV	No	Ref	Ref		
	Yes	1.178 ( 0.566 - 2.451 )	0.662		
AJCC Grade	I	Ref	Ref		
	II	1.248 ( 0.52 - 2.994 )	0.62		
	III	0.657 ( 0.259 - 1.662 )	0.375		
HBV	No	Ref	Ref	Ref	Ref
	Yes	10.471 ( 2.148 - 51.042 )	0.004	25.684 ( 1.168 - 54.832 )	0.04
Liver cirrhosis	No	Ref	Ref		
	Yes	1.143 ( 0.511 - 2.556 )	0.745		
Lymphatic metastasis	No	Ref	Ref	Ref	Ref
	Yes	5.792 ( 2.45 - 13.694 )	<0.001	12.288 ( 3.2 - 47.195 )	<0.001
Microvascular invasion	No	Ref	Ref	Ref	Ref
	Yes	2.932 ( 1.442 - 5.961 )	0.003	3.292 ( 1.316 - 8.234 )	0.011
Satellite lesions	No	Ref	Ref		
	Yes	1.849 ( 0.902 - 3.791 )	0.093		
Tumor thrombus	No	Ref	Ref		
	Yes	1.32 ( 0.607 - 2.871 )	0.483		
Tumor number	Single tumor	Ref	Ref		
	Multiple tumors	1.055 ( 0.515 - 2.161 )	0.884		
Tumor size		1.047 ( 0.961 - 1.141 )	0.293		
RBC		1.053 ( 0.476 - 2.33 )	0.899		
Hb		0.994 ( 0.972 - 1.016 )	0.573		
PLT		1.004 ( 0.999 - 1.009 )	0.155		
WBC		1.103 ( 0.993 - 1.226 )	0.068		
Neutrophil		1.123 ( 1.016 - 1.24 )	0.022	0.828 ( 0.69 - 0.994 )	0.043
Lymphocyte		0.834 ( 0.492 - 1.414 )	0.501		
Macrophage		1.647 ( 0.251 - 10.791 )	0.603		
TBil		0.988 ( 0.922 - 1.057 )	0.72		
ALB		0.881 ( 0.804 - 0.965 )	0.007	0.897 ( 0.771 - 1.043 )	0.157
ALT		1.002 ( 1 - 1.003 )	0.072		
AST		1.004 ( 1 - 1.009 )	0.039	1.005 ( 1 - 1.01 )	0.064
ALP		1.002 ( 0.997 - 1.007 )	0.439		
GGT		1.163 ( 0.997 - 1.392 )	0.083		
CEA		1.059 ( 1.017 - 1.102 )	0.005	1.107 ( 1.014 - 1.208 )	0.024
CA19-9		1.001 ( 1 - 1.002 )	0.047	1.001 ( 0.997 - 1.003 )	0.112
PT		1.75 ( 1.364 - 2.246 )	<0.001	1.657 ( 1.059 - 2.594 )	0.027
FIB		1.248 ( 0.899 - 1.731 )	0.185		

**Abbreviations:** RBC, red blood cell; Hb, hemoglobin; PLT, platelets; WBC, white blood cell; TBil, total bilirubin; ALB, albumin; ALP, alkaline phosphatase; GGT, gamma-glutamyl transpeptidase; PT, prothrombin time; FIB, fibrinogen; CA19-9, carbohydrate antigen 19-9; GAR, gamma-glutamyl transpeptidase to albumin ratio.

## Abbreviations

cHCC-CCA: combined hepatocellular carcinoma and cholangiocarcinoma
ROC: Receiver operating characteristic curves
MVI: microvascular invasion
GAR: gamma-glutamyl transpeptidase to albumin ratio
CEA: carcinoembryonic antigen
PT: prothrombin time
AFP: alpha-fetoprotein
HBV: hepatitis B virus
AJCC: American Joint Committee on Cancer
RBC: red blood cell
WBC: white blood cell
Hb: hemoglobin
PLT: platelet
TBil: total bilirubin
ALB: albumin
AST: aspartate aminotransferase
ALT: alanine aminotransferase
CA19-9: carbohydrate antigen 19-9
ALP: alkaline phosphatase
GGT: gamma-glutamyl transpeptidase
FIB: plasma fibrinogen
OS: overall survival
